# Retraction: Walach et al. The Safety of COVID-19 Vaccinations—We Should Rethink the Policy. *Vaccines* 2021, *9*, 693

**DOI:** 10.3390/vaccines9070729

**Published:** 2021-07-02

**Authors:** Vaccines Editorial Office

**Affiliations:** MDPI, St. Alban-Anlage 66, 4052 Basel, Switzerland; vaccines@mdpi.com

The journal retracts the article, The Safety of COVID-19 Vaccinations—We Should Rethink the Policy [1], cited above. 

Serious concerns were brought to the attention of the publisher regarding misinterpretation of data, leading to incorrect and distorted conclusions. 

The article was evaluated by the Editor-in-Chief with the support of several Editorial Board Members. They found that the article contained several errors that fundamentally affect the interpretation of the findings. 

These include, but are not limited to: 

The data from the Lareb report (https://www.lareb.nl/coronameldingen) in The Netherlands were used to calculate the number of severe and fatal side effects per 100,000 vaccinations. Unfortunately, in the manuscript by Harald Walach et al. these data were incorrectly interpreted which led to erroneous conclusions. The data was presented as being causally related to adverse events by the authors. This is inaccurate. In The Netherlands, healthcare professionals and patients are invited to report suspicions of adverse events that may be associated with vaccination. For this type of reporting a causal relation between the event and the vaccine is not needed, therefore a reported event that occurred after vaccination is not necessarily attributable to vaccination. Thus, reporting of a death following vaccination does not imply that this is a vaccine-related event. There are several other inaccuracies in the paper by Harald Walach et al. one of which is that fatal cases were certified by medical specialists. It should be known that even this false claim does not imply causation, which the authors imply. Further, the authors have called the events ‘effects’ and ‘reactions’ when this is not established, and until causality is established they are ‘events’ that may or may not be caused by exposure to a vaccine. It does not matter what statistics one may apply, this is incorrect and misleading.

The authors were asked to respond to the claims, but were not able to do so satisfactorily. The authors were notified of the retraction and did not agree. 
